# Green MRI for neuroimaging: monitoring brain tumors using non-contrast sequences

**DOI:** 10.1007/s11604-026-02045-y

**Published:** 2026-07-10

**Authors:** Kazuhiro Tsuchiya, Miho Gomyo, Shichiro Katase, Kenichi Yokoyama

**Affiliations:** Department of Radiology, Kyorin University School of Medicine, 6-20-2 Shinkawa, Mitaka City, Tokyo 181-8611 Japan

**Keywords:** Green radiology, MRI, Brain tumor, Gadolinium-based contrast agents (GBCAs), Arterial spin labeling (ASL)

## Abstract

The emerging concept of "Green Radiology" aims to mitigate the environmental impact of medical imaging while maintaining high standards of patient care. Among various modalities, MRI is particularly resource-intensive. This review focuses on the clinical feasibility and significance of minimizing gadolinium-based contrast agent (GBCA) administration in neuroimaging, specifically for the longitudinal follow-up of brain tumors. Key points of this review are as follows: (1) Rationales for "Green" MRI: Beyond patient safety concerns such as adverse reactions and gadolinium retention in the brain and bone, GBCAs pose significant environmental risks due to their persistence in global water systems and potential eco-toxicity. (2) Feasibility in extra-axial tumors: For many extra-axial lesions, including meningiomas, schwannomas, and macro-PitNETs (≥ 10 mm), non-contrast MRI sequences—leveraging the inherent contrast of cerebrospinal fluid—provide sufficient diagnostic information for routine surveillance. (3) Strategies for intra-axial tumors: While GBCAs remain the gold standard for high-grade gliomas and metastases, a "non-contrast-first" strategy may be viable for tumors with indolent progression. In these cases, GBCAs can be reserved for instances where interval changes are first identified on unenhanced sequences. (4) Advanced contrast-free alternatives: Non-invasive techniques such as arterial spin labeling (ASL), chemical exchange saturation transfer (CEST) imaging, and advanced diffusion-weighted imaging (DWI) offer powerful metabolic and microstructural insights without the need for chemical agents. (5) Future perspectives: Deep learning-based technologies, including "virtual contrast" synthesis and dosage reduction algorithms, hold immense potential to harmonize diagnostic excellence with environmental sustainability. Transitioning toward "Green Neuroradiology" through the judicious use of GBCAs and the adoption of advanced non-contrast sequences is a practical and necessary step for sustainable radiological practice. This review highlights the concept of "Green MRI" for sustainable neuroimaging. We discuss the clinical and environmental rationales for minimizing the use of GBCAs, particularly in the follow-up of extra-axial tumors. Furthermore, we explore how advanced sequences and deep learning provide viable, contrast-free alternatives for future radiological practice.

## Introduction

Green magnetic resonance imaging (MRI) represents a paradigm shift toward environmentally sustainable radiology. It encompasses practices aimed at minimizing energy consumption, waste, and resource depletion without compromising diagnostic performance or patient safety. Despite its clinical utility, MRI remains resource-intensive, facing several sustainability challenges: high power consumption (particularly for cooling superconducting magnets), reliance on helium as a non-renewable resource, significant idle energy consumption, and a substantial environmental footprint for MRI suite infrastructure, including specialized shielding and cooling systems. While addressing infrastructure and helium constraints requires long-term technological and systemic shifts, the routine use of gadolinium-based contrast agents (GBCAs) presents an immediate area for clinical intervention due to growing ecological and clinical concerns.

To provide a practical roadmap for sustainable practice, this narrative review specifically addresses the use of GBCAs in the longitudinal follow-up of brain tumors—a clinical scenario where contrast enhancement has long been considered the gold standard. While acknowledging the broader scope of Green MRI, we focus on GBCA reduction as a highly actionable and impactful target in current neuroradiological workflows. In this context, we explore the following key questions:

1. What are the primary rationales for minimizing GBCA administration?
2. Are GBCAs indispensable for the follow-up of brain tumors? We provide illustrative cases from our experience where non-contrast imaging provided sufficient diagnostic information.
3. What are the current limitations of "Green MRI" in neuro-oncology, and what are the future perspectives?

## Basic concepts of “Green” MRI

### What is “Green” MRI?

Green radiology is an emerging paradigm aimed at mitigating the environmental impact of medical imaging while maintaining high standards of care. This concept has gained significant traction in response to the escalating global climate crisis [1–7]. Within this framework, "Green MRI" refers to environmentally conscious practices designed to minimize energy consumption, waste, and resource depletion without compromising diagnostic performance or patient safety. As previously noted, MRI presents several unique sustainability challenges. This article specifically focuses on strategies for minimizing the use of GBCAs and discusses critical considerations for both radiologists and referring physicians.

### Minimizing use of GBCAs

Several factors necessitate a more judicious use of GBCAs. It is increasingly recognized that limiting contrast administration to cases of clear clinical necessity is not only safer for the patient but also more environmentally responsible. This responsibility extends beyond mitigating downstream water contamination; it also encompasses "upstream" sustainability challenges. Gadolinium is a strategic, geographically concentrated rare-earth element, and its energy-intensive mining and refining processes are associated with significant environmental burdens. By reducing clinical reliance on GBCAs, the radiology community can minimize the total environmental footprint of these agents throughout their entire life cycle—from extraction to disposal.

### Adverse effects of GBCAs

The spectrum of GBCA-related adverse reactions ranges from mild symptoms, such as urticaria, nausea, and localized heat sensation, to severe events including seizures and anaphylaxis. A major historical and clinical concern is nephrogenic systemic fibrosis (NSF) [8–11]. Characterized by skin thickening and joint contractures, NSF is triggered by the dissociation of free ions from their chelates, occurring more frequently with linear GBCAs than with macrocyclic agents. While strict restrictions were historically placed on GBCA use in patients with renal impairment, regulations in the United States have recently been relaxed [12]. This shift reflects the dramatic decline in NSF incidence following the transition from linear to more stable macrocyclic GBCAs.

### Gadolinium retention in the human body

Gadolinium can accumulate in the body even in individuals with normal renal function. Although the clinical impact of these deposits remains a subject of ongoing investigation, gadolinium retention in the brain is well-documented as high signal intensity in the globus pallidus and dentate nucleus on T1-weighted images [13–15]. Notably, the highest concentration of deposited gadolinium is found in bone tissue [16]. While brain retention is primarily associated with linear GBCAs, accumulation in the skeletal system occurs with both linear and macrocyclic agents. Autopsy studies have further demonstrated that gadolinium can persist in these tissues for years after even a single administration [16, 17].

Despite the lack of proven causality, recent epidemiological data warrant careful consideration. A nationwide population-based cohort study from Korea reported a potential association between exposure to certain GBCAs and an increased risk of parkinsonism [18]. While these findings reinforce the need for continued vigilance, potential confounding factors and biases inherent in large-scale studies must be considered. Overall, this emerging evidence supports a balanced interpretation while further justifying the "Green MRI" principle of minimizing unnecessary contrast administration.

### Environmental contamination

Environmental contamination occurs primarily through the excretion of GBCAs in urine, which then enters municipal wastewater systems. Removal rates in conventional sewage treatment plants are reportedly less than 10%, leading to the detection of gadolinium in tap water worldwide. Bioaccumulation has been confirmed in various aquatic organisms, including daphnids, bivalves, and fish [19–21]. Furthermore, research has indicated detrimental effects on gene expression in bivalves and skeletal development in sea urchins [22, 23]. Extensive contamination of seawater and river systems has been documented globally [24–27].

## Is contrast administration essential for brain tumor follow-up?

### Significance of GBCA administration in MRI of brain tumors

GBCAs enhance lesion detection and characterization by highlighting blood–brain barrier (BBB) disruption and tumor-associated neoangiogenesis. For intra-axial tumors, such as gliomas, lymphomas, and metastases, GBCAs are instrumental in delineating tumor extent, identifying high-grade components, and monitoring treatment response. In extra-axial tumors, contrast enhancement aids in differential diagnosis and precise preoperative mapping of the relationship between the tumor and adjacent vascular structures.

### Evaluation of extra-axial tumors without GBCAs

Growing evidence suggests that many extra-axial tumors can be monitored effectively using non-contrast MRI. The inherent contrast provided by the surrounding cerebrospinal fluid (CSF) often allows for clear margin delineation.

**Fig. 1:**
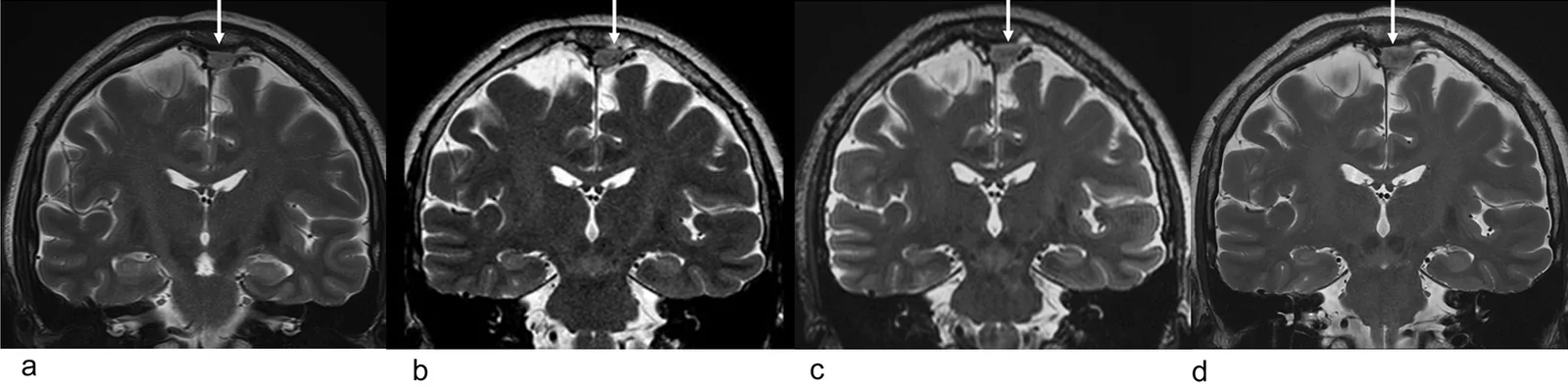
T2-weighted coronal images from a woman with parasagittal meningioma (not histologically proven) obtained during her 50 s and 60 s at 4 occasions during the follow-up (July 201X [**a**], June 201X + 3 [**b**], August 201X + 7 [**c**], and August 201X + 9 [**d**]). This patient was allergic to GBCAs, which has led her to be followed up exclusively by non-contrast MRI. These serial T2-weighted images over 9 years show that her tumor is almost stable in size and signal (arrow)

**Fig. 2:**
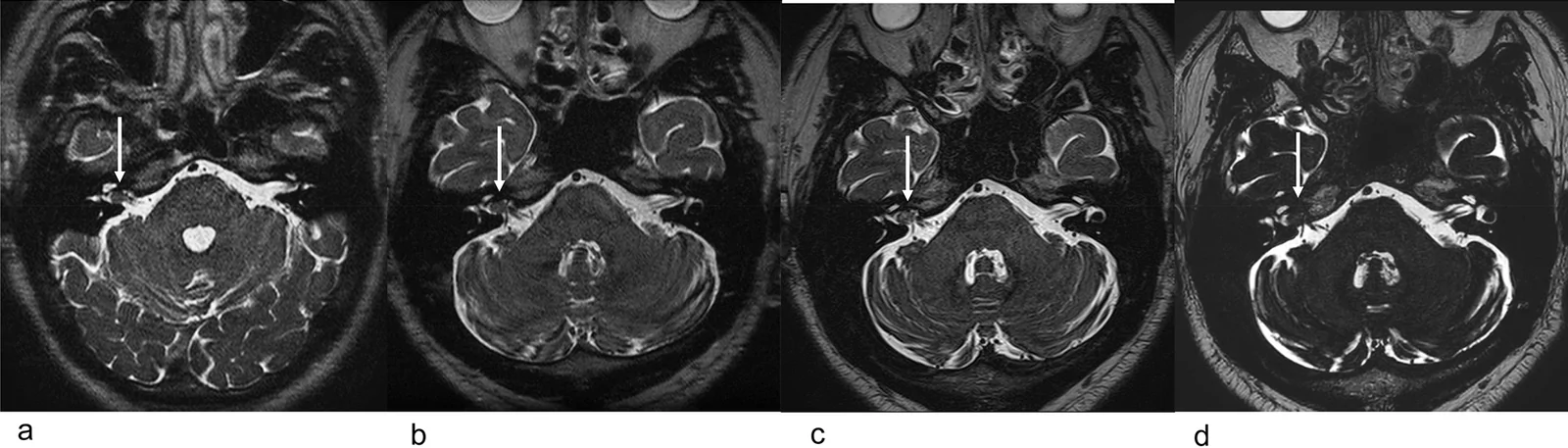
MR cisternographic images from a man with acoustic schwannoma (not histologically proven) acquired during his 40 s and 50 s at four occasions during follow-up (December 201X [**a**], October 201X + 6 [**b**], January 201X + 9 [**c**], and July 201X + 11 [**d**]). MR cisternography over more than 10 years reveals a gradual increase in tumor size (arrow)

**Fig. 3:**
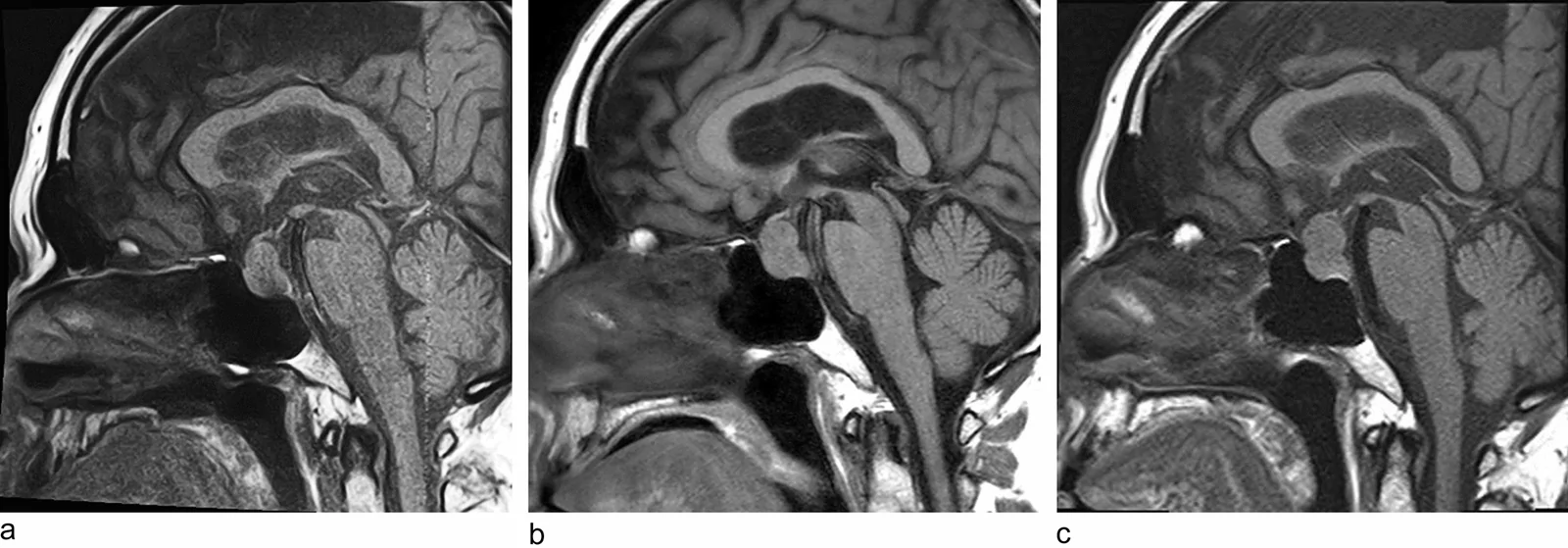
T1-weighted sagittal images from a man with PitNET (not histologically proven) obtained during her 70 s at 3 occasions during the follow-up (March 202X + 2 [**a**], March 201X + 3 [**b**], and August 202X + 4 [**c**]). These thin-slice serial non-contrast T1-weighted images demonstrate a gradual increase in tumor size

**Fig. 4:**
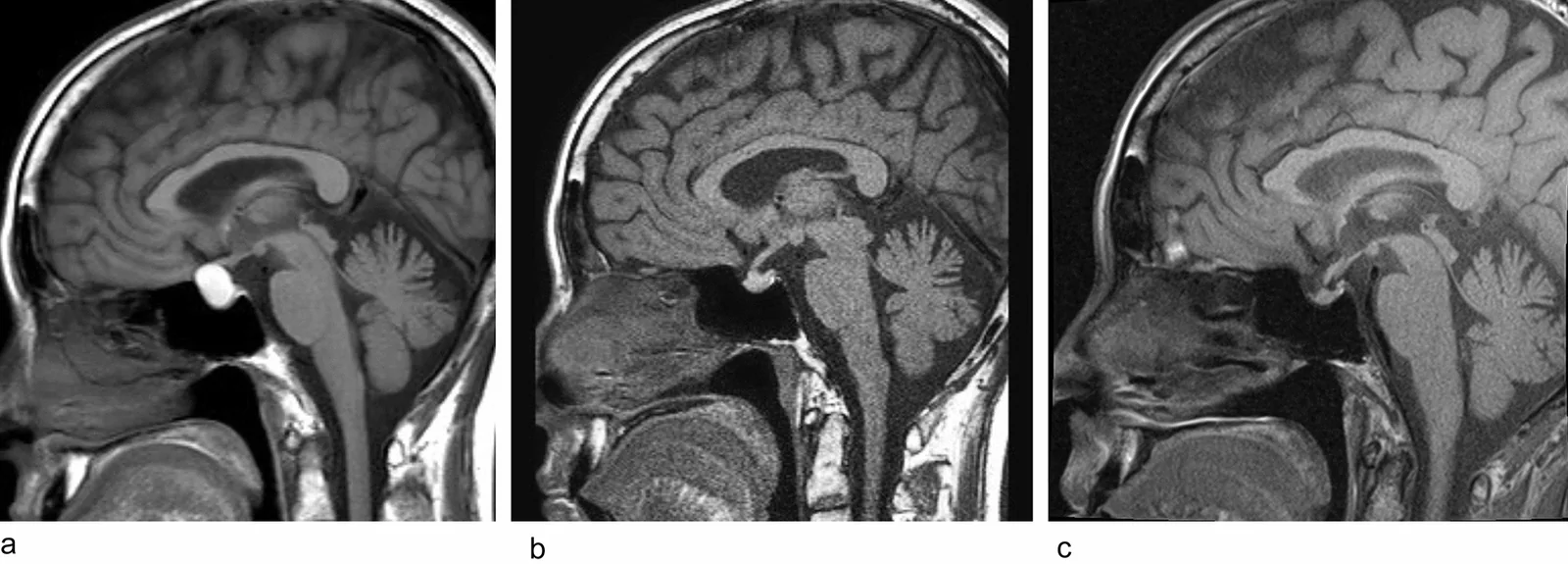
T1-weighted sagittal images from a woman with Rathke’s cleft cyst (not histologically proven) obtained during her 50 s at 3 occasions during the follow-up (October 201X [**a**], May 201X + 1 [**b**], and December 201X + 2 [**c**]). These three T1-weighted images show that the hyperintense tumor is gradually shrinking. Temporal changes in the tumor can sometimes be detected by sequential evaluation of imaging techniques other than high-resolution T1-weighted images, which may produce characteristic signals

**Fig. 5:**
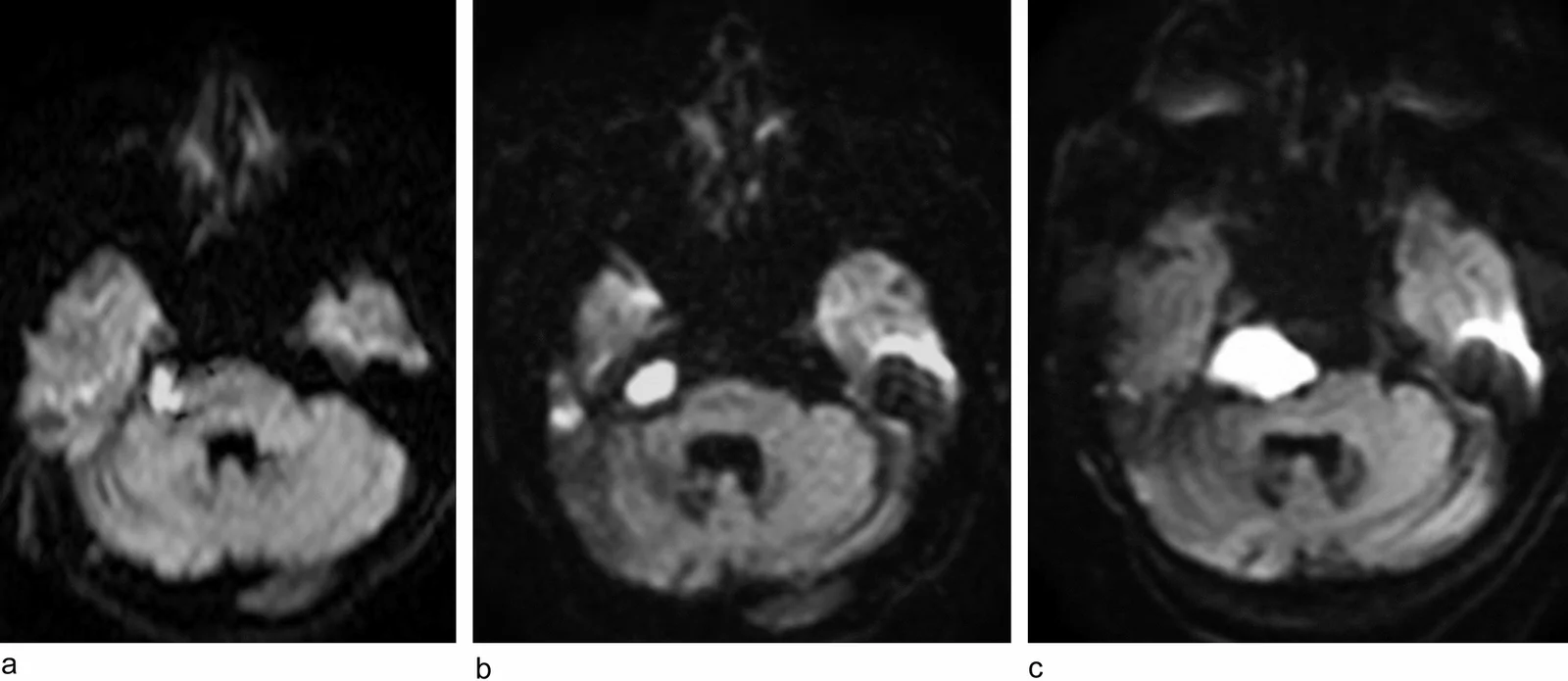
Diffusion-weighted images from a man with post-operative cerebellopontine-angle epidermoid cyst acquired at 3 occasions during his 60 s to 80 s (March 200X [**a**], September 200X + 10 [**b**], and September 200X + 19 [**c**]). Epidermoid cysts can be effectively followed up using diffusion-weighted imaging, which shows them with a clear hyperintensity. In this case, a gradual increase in the size of the post-operative residual of the epidermoid cyst is clearly demonstrated

- *Meningiomas* Current European (EANO) and Danish guidelines already suggest T2-weighted imaging (T2WI) for routine follow-up [28–31] **(**Fig. 1**)**. While skull base lesions remain challenging, convexity or parasagittal meningiomas are easily assessed without GBCAs. Recent data confirm that non-contrast T2WI is non-inferior to contrast-enhanced T1-weighted imaging for detecting tumor growth [32].
- *Schwannomas* In clinical practice, the detection and surveillance of vestibular schwannomas rely heavily on non-contrast, high-resolution 3D T2-weighted sequences (e.g., CISS, FIESTA, or SPACE), which provide excellent diagnostic performance **(**Fig. 2**)**. However, contrast-enhanced T1-weighted imaging remains necessary for preoperative planning to assess internal architecture and vascularity.
- *Pituitary Lesions* Macro-PitNETs (≥ 10 mm) can be adequately monitored using high-spatial-resolution non-contrast MRI targeted to the sella **(**Fig. 3**)**. In contrast, the detection of micro-Pit Nets (< 10 mm) still necessitates dynamic contrast-enhanced MRI.
- *Other Lesions* The imaging characteristics of certain extra-axial pathologies, such as cavernous sinus hemangiomas and epidermoid cysts, render them amenable to non-contrast monitoring (Figs. 4, 5). For instance, epidermoid cysts are typically characterized by markedly restricted diffusion on diffusion-weighted imaging (DWI), which often provides superior diagnostic clarity for detecting small residual or recurrent lesions compared to contrast-enhanced T1-weighted images. Similarly, cavernous sinus hemangiomas exhibit a pathognomonic "light bulb" hyperintensity on T2-weighted images, allowing for reliable longitudinal assessment of tumor morphology and size without the need for periodic GBCA administration.

### Limitations in intra-axial tumor imaging

Relying solely on non-contrast MRI for gliomas, lymphomas, and metastases remains challenging. The following are some of the main limitations that reflect the fact that the use of GBCA does not solve all diagnostic problems:

- Suboptimal delineation of infiltrative margins.
- Lower sensitivity for detecting small lesions or early-stage recurrences.
- Difficulty in distinguishing tumor progression from treatment-related changes (e.g., pseudoprogression).
- Lack of information regarding tumor grading and biological aggressiveness.

Nonetheless, a 'Green' approach may be feasible by reserving GBCAs for cases where changes are first detected on non-contrast sequences. This is supported by evidence that T2/FLAIR volumetric changes often precede the development of new contrast enhancement in glioma surveillance [33]. Furthermore, since ADC values derived from DWI serve as established surrogate markers for cellularity and malignant transformation [34, 35], these sequences provide critical insights into biological aggressiveness without the need for routine GBCA administration.

### GBCA use in perfusion imaging of brain tumors

While dynamic susceptibility contrast (DSC) perfusion is a long-standing standard, arterial spin labeling (ASL)—a gadolinium-free technique—has shown comparable diagnostic performance in evaluating tumor hemodynamics [36–38]. ASL is less susceptible to metal or hemorrhage-induced artifacts than DSC, making it particularly valuable for postoperative assessment [38]. In the context of "Green MRI," ASL is a vital tool for assessing metabolic activity without chemical waste.

### Recommended sequences to follow up extra-axial tumors

Based on institutional experience and established literature, we propose a standardized set of sequences for the non-contrast follow-up of extra-axial tumors **(**Table 1**)**.

**Table 1:** Recommended sequences for the follow-up of extra-axial tumors based on our experience

	Recommended	Optional	Comments	Representative cases
Meningiomas	T2-weighted imaging		Adjust field-of-view and slice thickness depending on the lesion	Figure 1
Acoustic schwannomas	3D heavily T2-weighted scans (MR cisternography)		Add reconstructed coronal images	Figure 2
Macro-PitNETs (≥ 10 mm) Rathke's cleft cysts	Thin-slice T1/T2-weighted imaging (around 2 mm)	3D heavily T2-weighted scans (MR cisternography)		Figures 3, 4
Epidermoid cysts	Thin-slice diffusion-weighted imaging (around 2 mm)	3D heavily T2-weighted scans (MR cisternography)		Figure 5

## Emerging strategies and innovations for reducing GBCA exposure

To realize the vision of "Green Neuroradiology," several innovative approaches are being explored to minimize or even eliminate the need for traditional GBCA doses. These can be categorized into computational intelligence and chemical advancements.

### Deep learning and artificial intelligence

Deep learning (DL) has emerged as a powerful tool for dose optimization. Recent studies have demonstrated that convolutional neural networks (CNNs) can reconstruct high-quality, standard-dose-equivalent images from low-dose acquisitions (e.g., 10%–25% of the standard dose) by effectively reducing noise and enhancing image contrast [39]. Furthermore, the concept of "virtual contrast enhancement" is gaining attention. By utilizing multi-parametric non-contrast sequences (such as T1, T2, FLAIR, and DWI) as input, DL models can synthesize T1-weighted contrast-like images without any actual GBCA administration [40]. While these technologies require further rigorous clinical validation to ensure the absence of "hallucinations" or missed micro-lesions, they represent a significant frontier in reducing the environmental and biological burden of gadolinium.

### Next-generation contrast agents

Parallel to computational efforts, advancements in contrast agent chemistry offer promising alternatives. High-relaxivity GBCAs are already in clinical use, allowing for a lower molar dose of gadolinium while maintaining diagnostic image quality. Beyond gadolinium, manganese-based contrast agents (MBCAs) are being actively investigated. As a paramagnetic ion that handles biological pathways differently than gadolinium, manganese provides effective T1 shortening and may circumvent concerns regarding long-term brain retention and environmental accumulation [41]. Although currently in various stages of clinical trials, these agents signify a paradigm shift toward more biologically inert contrast-enhanced imaging.

## Conclusion

It is important to acknowledge that GBCAs remain indispensable in specific scenarios. Nevertheless, the clinical landscape is evolving. ASL has already emerged as a robust, contrast-free alternative for perfusion imaging. Beyond perfusion, other non-invasive molecular imaging techniques—including chemical exchange saturation transfer (CEST), MR spectroscopy, and advanced diffusion-weighted imaging—provide critical insights into metabolic activity, cellularity, and microstructural integrity. Additionally, the integration of DL offers a transformative path forward. Recent advances suggest that artificial intelligence (AI) can significantly reduce required contrast dosages or even synthesize "virtual" contrast-enhanced T1-weighted images without any GBCA administration [39, 42–44]. Future perspectives in contrast agent chemistry include the clinical translation of lower gadolinium content or high-relaxivity GBCAs, which could allow for significant dose reductions while maintaining diagnostic image quality. Furthermore, the development of non-gadolinium-based contrast agents, such as MBCAs, is gaining traction as a means to circumvent both environmental and retention concerns. Although these novel agents and formulations are currently in various stages of pre-clinical and clinical investigation, they represent a fundamental shift toward a more sustainable and biologically inert future for contrast-enhanced neuroradiology.
